# Study of matrix metalloproteinases and their inhibitors in breast cancer

**DOI:** 10.1038/sj.bjc.6603666

**Published:** 2007-03-06

**Authors:** F J Vizoso, L O González, M D Corte, J C Rodríguez, J Vázquez, M L Lamelas, S Junquera, A M Merino, J L García-Muñiz

**Affiliations:** 1 Servicio de Cirugía General, Hospital de Jove, Gijón, Spain; 2 Unidad de Investigación, Hospital de Jove, Gijón, Spain; 3 Instituto Universitario de Oncología del Principado de Asturias, Oviedo, Spain; 4 Servicio de Anatomía Patológica, Hospital de Jove, Gijón, Spain; 5 Servicio de Ginecología, Hospital de Jove, Gijón, Spain; 6 Servicio de Anatomía Patológica, Hospital de Cabueñes, Gijón, Spain; 7 Servicio de Cirugía General, Hospital Universitario Central de Asturias, Oviedo, Spain

**Keywords:** breast cancer, tissue arrays, prognosis, MMP, TIMP, tumoural invasion, metastasis

## Abstract

An immunohistochemical study was performed using tissue microarrays and specific antibodies against matrix metalloproteinases (MMPs) 1, 2, 7, 9, 11, 13, 14, and their tisullar inhibitors (TIMPs) 1, 2, and 3. More than 2600 determinations on cancer specimens from 131 patients with primary ductal invasive tumours of the breast (65 with and 66 without distant metastasis) and controls were performed. Staining results were categorised using a score based on the intensity of the staining and a specific software program calculated the percentage of immunostained cells automatically. We observed a broad variation of the total immunostaining scores and the cell type expressing each protein. There were multiple and significant associations between the expression of the different MMPs and TIMPs evaluated and some parameters indicative of tumour aggressiveness, such as large tumour size, advanced tumour grade, high Nottinham prognostic index, negative oestrogen receptor status, peritumoural inflammation, desmoplastic reaction, and infiltrating tumoural edge. Likewise, the detection of elevated immunohistochemical scores for MMP-9, 11, TIMP-1, and TIMP-2, was significantly associated with a higher rate of distant metastases. The expression of MMP-9 or TIMP-2 by tumour cells, MMP-1, 7, 9, 11, 13, or TIMP-3 by fibroblastic cells, and MMP-7, 9, 11, 13, 14, TIMP-1, or TIMP-2 by mononuclear inflammatory cells, was also significantly associated with a higher rate of distant metastases.

Relapse in the form of metastases within 5 years of surgery occurs in about half the women with primary breast cancer with originally apparently localised tumours. However, it is difficult to predict this event because breast cancer is a heterogeneous disease encompassing a variety of pathological entities and a wide range of clinical behaviours, even in patient groups that appear to be clinically similar. Therefore, and despite having a number of classical prognostic variables available, new prognostic factors should be identified to improve the present risk classification and thereby to develop a more rational management of breast cancer patients.

Tumour invasion and metastasis development are the primary determinants of patient outcome and, accordingly, molecules involved in these processes are obvious candidates to be identified as new prognostic markers in breast cancer. Degradation of the stromal connective tissue and basement membrane components are key elements in tumour invasion and metastasis. Proteolytic enzymes of various classes execute the breaking down of matrix elements. However, some components, particularly the interstitial collagens, are very resistant to proteolytic attacks, being degraded only by matrix metalloproteinases (MMPs) (Nelson *et al*, 2000). The human MMP family currently consists of 28 members of homologous zinc-dependent endopeptidases that can be divided into eight structural classes or, on the basis of their substrate specificity and primary structure, into the more familiar subgroups of collagenases (MMP-1, 8, and 13), gelatinases (MMP-2 and 9), stromelysins (MMP-3, 10, and 11), membrane-associated MMPs (MMP-14, 15, 16, 17, 23, 24, and 25), and other novel MMPs (Brinckerhoff *et al*, 2000; Overall and Lopez-Otin, 2002; Demers *et al*, 2005). Matrix metalloproteinases are synthesised as inactive zymogens, which are then activated predominantly pericellularly either by other MMPs or by serine proteases. The activity of MMPs is specifically inhibited by the so-called tissue inhibitors of metalloproteases (tisullar inhibitors (TIMPs)). Currently, four different TIMPs are known to exist: TIMPs 1, 2, 3, and 4.

There are available data clearly challenging the classic dogma stating that MMPs promote metastases exclusively by modulating the remodelling of extracellular matrix, as MMPs able to impact *in vivo* on tumour cell behaviour as a consequence of their ability to cleave growth factors, cell surface receptors, cell adhesion molecules, or chemokines/cytoquines have also been identified (Manes *et al*, 1999; Noe *et al*, 2001; Egeblad and Werb, 2002; Turk *et al*, 2004). Furthermore, by cleaving proapoptotic factors, MMPs are able to produce a more aggressive phenotype via generation of apoptotic resistant cells (Fingleton *et al*, 2001). Matrix metalloproteinases may also regulate cancer/related angiogenesis, both positively through their ability to mobilise or activate proangiogenic factors (Stetler-Stevenson, 1999) and negatively via generation of angiogenesis inhibitors, such as angiostatin and endostatin, cleaved from large protein precursors (Cornelius *et al*, 1998). In addition, it is now assumed that TIMPs are multifactorial proteins also involved in the induction of proliferation and the inhibition of apoptosis (Jiang *et al*, 2002; Wurtz *et al*, 2005).

The objectives of the present work were to evaluate the morphological expression and clinical relevance of several MMPs and TIMPs of biological importance in invasive ductal carcinomas of the breast, by using the tissue microarray (TMA) technique, which has allowed us to process a large number of tissue specimens for a wide range of protein determinations (Kononen *et al*, 1998; Camp *et al*, 2000).

## MATERIALS AND METHODS

### Patients' selection, patients' characteristics, and tissue specimen handling

This study comprised 131 women with a histologically confirmed diagnosis of early breast cancer and treated between 1990 and 2001. We selected women with the following inclusion criteria: invasive ductal carcinoma, at least 10 histopathologicallyassessed axillary lymph nodes, and a minimum of 5 years of follow-up in those women without tumoral recurrence. The exclusion criteria were the following: metastatic disease at presentation, prior history of any type of malignant tumour, bilateral breast cancer at presentation, having received any type of neoadjuvant therapy, development of loco-regional recurrence during the follow-up period, development of a second primary cancer, and absence of sufficient tissue in the paraffin blocks used for manufacturing the TMAs. From a total of 1053 patients fulfilling these criteria, we selected randomly a sample size of 131 patients, in accordance with four different groups of similar size and stratified with regard to nodal status and with the development of metastatic disease, which were the key measure variables of the study. Thus, we include an important number of events in both node-negative and node-negative patient subgroups (half the cases with distant metastasis during the follow-up period in each one of these subgroups) for securing the statistical power of the survival analysis. Patients' characteristics included in the two main groups, with or without distant metastases, are listed in Table 1. Nottingham prognostic grade was assessed in accordance with Galea (1992).

Women were treated according to the guidelines used in our institution. The study adhered to national regulations and was approved by our institution's Ethics and Investigation Committee. The end point was distant metastatic relapse. The median follow-up period in patients without metastasis was 87.5 months, and 52.7 months in patients with metastasis.

### Tissue microarrays and immunohistochemistry

Routinely fixed (overnight in 10% buffered formalin), paraffin-embedded tumour samples stored in our pathology laboratory files were used in this study. Histopathologically representative tumour areas were defined on haematoxylin and eosin (H&E)-stained sections and marked on the slide. Tumour tissue array blocks were obtained by punching a tissue cylinder (core) with a diameter of 1.5 mm through a histologically representative area of each ‘donor’ tumour block, which was then inserted into an empty ‘recipient’ tissue array paraffin block using a manual tissue arrayer (Beecker Instruments, Sun Praerie, Winconsin, USA) as described elsewhere (Parker *et al*, 2002). Collection of tissue cores was carried out under highly controlled conditions. Areas of non-necrotic cancerous tissue were selected for arraying by two experienced pathologists (LO González and AM Merino). Two cores were employed for each case. From the 131 tumour samples available, four tissue array blocks were prepared, each containing 33 tumour samples, as well as internal controls including four normal breast tissue samples from two healthy women that underwent reductive mammary surgery.

Four composite high-density TMA blocks were designed, and serial 5 *μ*m sections were consecutively cut with a microtome (Leica Microsystems GmbH, Wetzlar, Germany) and transferred to adhesive-coated slides. One section from each tissue array block was stained with H&E, and these slides were then reviewed to confirm that the sample was representative of the original tumour. Immunohistochemistry was carried out on these sections of TMA fixed in 10% buffered formalin and embedded in paraffin using a TechMate TM50 autostainer (Dako, Glostrup, Denmark). Antibodies for MMPs and TIMPs were obtained from Neomarker (Lab Vision Corporation, Fremont, CA, USA). The dilution for each antibody was established based on negative and positive controls (1/50 for MMP-2, 7, and 14, TIMP-2 and 3; 1/100 for MMP-1, 9, and 13 and TIMP-1; and 1/200 for MMP-11).

Tissue sections were deparaffinised in xylene, and then rehydrated in graded concentrations of ethyl alcohol (100, 96, 80, and 70%, then water). To enhance antigen retrieval only for some antibodies, TMA sections were microwave-treated (H2800 Microwave Processor, EBSciences, East Granby, Connecticut, USA) in citrate buffer (Target Retrieval Solution, Dako) at 99°C for 16 min. Endogenous peroxidase activity was blocked by incubating the slides in peroxidase-blocking solution (Dako) for 5 min. The EnVision Detection Kit (Dako) was used as the staining detection system. Sections were counterstained with haematoxilin, dehydrated with ethanol, and permanently coverslipped.

### TMA analysis

For each antibody preparation studied, the location of immunoreactivity, percentage of stained cells, and intensity were determined. All the cases were semiquantified for each protein-stained area. An image analysis system with the Olympus BX51 microscope and analysis soft (analySIS®, Soft imaging system, Münster, Alemania) was employed as follows: tumour sections were stained with antibodies according to the method explained above and counterstained with haematoxilin. There are different optical thresholds for both stains. Each core was scanned with a × 400 power objective in two fields per core. Fields were selected searching for the protein-stained areas. The computer program selects and traces a line around antibody-stained areas (higher optical threshold: red spots), with the remaining, non-stained areas (haematoxilin-stained tissue with lower optical threshold) standing out as a blue background. Any field has an area ratio of stained (red) *vs* non-stained areas (blue). A final area ratio was obtained after averaging two fields. To evaluate immunostaining intensity we used a numeric score ranging from 0 to 3, reflecting the intensity as follows: 0, no staining; 1, weak staining; 2, moderate staining; and 3, intense staining. Using an Excel spreadsheet, the mean score was obtained by multiplying the intensity score (I) by the percentage of stained cells (PC) and the results were added together (total score: I × PC). This overall score was then averaged with the number of cores that were carried out for each patient. If there was no tumour in a particular core, then no score was given. In addition, for each tumour, the mean score of two core biopsies was calculated.

Furthermore, whole-tissue sections from tumoural blocks from a subset of 10 cases were compared with the corresponding TMA discs, regarding each MMP and TIMP expression. Those cases were selected randomly, and the obtained clinicopathological data were very similar to those from the whole series. Each whole-tissue section was scanned with a × 400 power lens in 10 different fields. Fields were selected searching for the protein-stained areas, as described above.

### Data analysis and statistical methods

Immunostaining score values for each protein were expressed as median (range). Comparison of immunostaining values between groups was made with the Mann–Whitney or Kruskall–Wallis tests. Statistical results were corrected applying Bonferroni's correction. For metastasis-free survival analysis, we used Cox's univariate method. Cox's regression model was used to examine interactions of different prognostic factors in a multivariate analysis. Expression profiles were analysed by the unsupervised hierarchical clustering method that organises proteins in a tree structure, on the basis of their similarity. Data were reformatted as follows: −3 designated negative staining, 3 positive staining, missing data was left blank. The score values were reformatted (positive–negative) choosing the median as cutoff value. We used the Cluster 3.0 program (average linkage, Pearson correlation). Results were displayed with Treeview (Eisen *et al*, 1998). The SPSS 11.5 program was used for all calculations.

## RESULTS

More than 2600 determinations in cancer specimens from 131 patients with primary invasive ductal carcinoma of the breast and controls were performed on TMAs. Minimal internal variance of score data between duplicate tissue cores from the same patients was detected in the tissue arrays, showing a high agreement for each protein (*r*>0.95 and *P*<0.0001). In the validation study there was total concordance in the global expression, as well as in the intensity of immunostaining, for each MMP and TIMP between TMA cases and the corresponding whole-tissue sections. In addition, there were highly significant correlations in the immunostaining scores between these two paired sets (*r*>0.90 and *P*<0.0001, for each protein).

**Figure 1:**
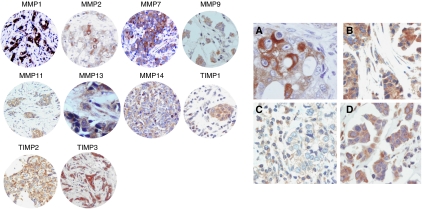
Left: examples of TMAs with immunostaining for each protein. 200 × Right: (**A**) immunohistochemical staining of MMP2 in epithelial cells, (**B**) TIMP3 in epithelial cells and fibroblastic cells, (**C**) TIMP3 in inflammatory mononuclear cells, and (**D**) TIMP2 in epithelial cells, fibroblast and inflammatory mononuclear cells. 400 × .

Figure 1 shows examples of TMAs with immunostaining for each protein evaluated. There was a wide variability in the immunostaining score values for each protein (Table 2). Immunostaining for all the proteins studied was localised predominantly in tumour cells, but also in stromal cells in a significant percentage of cases. There were significant associations between the total immunostaining scores for several proteins and clinicopathological parameters of tumoral aggressiveness (Table 2).

**Figure 2:**
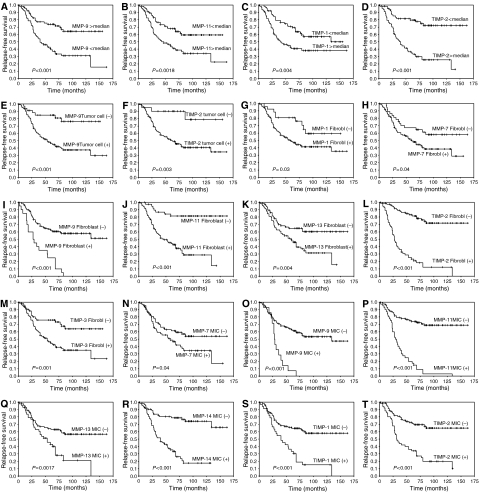
Kaplan–Meier survival curves as function a of the immunostaining score values of MMP-9 (**A**), MMP-11 (**B**), TIMP-1 (**C**), and TIMP-2 (**D**); expression by tumoral cells of MMP-9 (**E**) and TIMP-2 (**F**); expression by fibroblast cells of MMP-1 (**G**), MMP-7 (**H**), MMP-9 (**I**), MMP-11 (**J**), MMP-13 (**K**), TIMP-2 (**L**), and TIMP-3 (**M**); expression by mononuclear inflammatory cells of MMP-7 (**N**), MMP-9 (**O**), MMP-11 (**P**), MMP-13 (**Q**), MMP-14 (**R**), TIMP-1 (**S**), and TIMP-2 (**T**).

**Figure 3:**
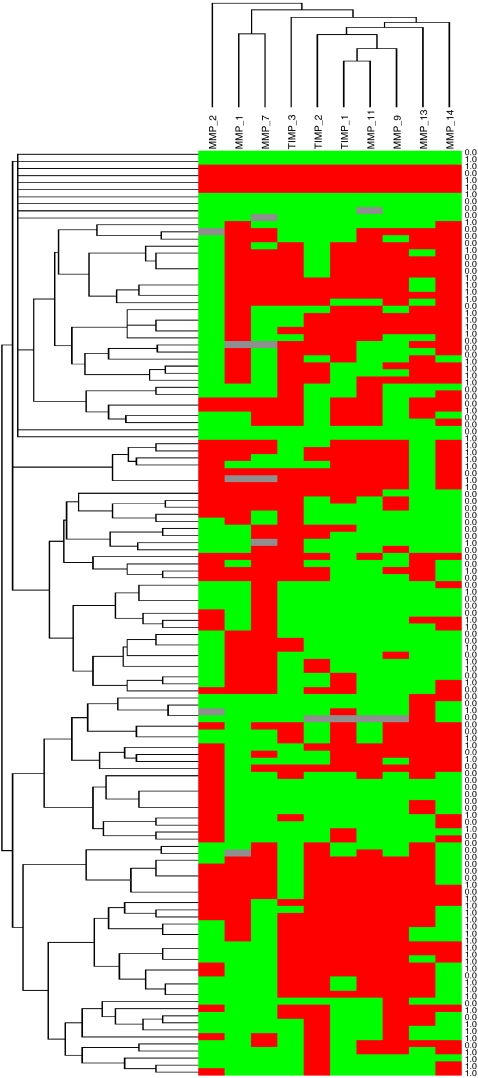
Graphical representation of two-dimensional unsupervised hierarchical clustering results on immunohistochemistry expression profiles of 10 proteins in 131 breast cancer samples. *Rows:* samples; *columns*, proteins. Protein expression scores are depicted according to a colour scale: red, positive staining; green, neative staining; grey, missing data. Dendogram of samples (to the left of matrix) and proteins (above matrix) represent overall similarities in expression profiles. *Status column*: 1=with recurrence; *0*, without recurrence, at the census point.

We initially investigated the possible association between the total immunostaining scores for each MMP and TIMP and the relapse-free survival, taking the median value of the immunostaining score for each protein as the cutoff point. Thus, we found that a high expression of MMP-9 and 11, TIMP-1 and 2 was significantly associated with a shortened relapse-free survival (Table 3 and Figure 2). In addition, our data showed that the expression of MMP-1, 7, 9, 11, 13, 14, TIMP-1, and 2, as a function of the cellular type (tumour cell, fibroblast, and/or inflammatory mononuclear cell) expressing the protein, was significantly associated with a shorter relapse-free survival (Table 3 and Figure 2). Additionally, to identify specific groups of tumours with distinct MMP/TIMP immunohistochemical expression profiles, the data were analysed by unsupervised hierarchical cluster analysis. The algorithm orders proteins on the horizontal axis and samples on the vertical axis based on similarity of their expression profiles. However, this did not produce a dendrogram with a well-defined cluster of tumours (Figure 3).

Multivariate analysis between classical prognostic factors according to Cox model demonstrated that tumoral stage (II: relative risk (RR) (confidence interval (CI))=1.8(0.9–3.6); III: 3.9(2–8); *P*<0.001) and PgR status (positive: 0.36(0.2–0.6), *P*<0.001) were significantly and independently associated with relapse-free survival. All the MMP and TIMP expressions that reached significance for predicting distant metastases in the univariate analysis were significantly and independently associated with relapse-free survival in the multivariate analysis (Table 3).

## DISCUSSION

This is, to the best of our knowledge, the first study analysing the expression of MMPs and TIMPs in human breast cancer by applying TMA technology, which allows one to integrate different biological aspects of the tumour in the morphological context of breast carcinoma.

Matrix metalloproteinases -2 (gelatinase A) and MMP-9 (gelatinase B) are related to tumour invasion and metastasis by their special capacity to degrade the type IV collagen found in basement membranes (Jones and Walker, 1997), and to induce angiogenesis (Egeblad and Werb, 2002). Our results are in accordance with those of previous reports showing that a high MMP-9 expression correlates significantly with tumoral aggressiveness and poor prognosis (Chantrain *et al*, 2004; Li *et al*, 2004; Pellikainen *et al*, 2004), as well as with other studies where high MMP-2 expression in carcinoma cells, in contrast, has been related to only a few inverse prognostic factors (Talvensaari-Mattila *et al*, 1998; Nakopoulou *et al*, 2003) or shown to have no association with clinicopathological parameters in breast cancer (Jones *et al*, 1999; Hirvonen *et al*, 2003; Talvensaari-Mattila *et al*, 2003). It has also been described that as breast cancer progresses, MMP-2 production increases during the early phases, whereas activation of MMP-9 occurs during the late cancerous stage (Liotta and Kohn, 2001), which could explain their different impact on prognosis in clinically detected invasive breast tumours.

Matrix metalloproteinase-1 (collagenase-1), the most ubiquitously expressed of the interstitial collagenases, is required for local invasion because it possesses the ability to efficiently degrade type I collagen – the principal component of connective tissue (Brinckerhoff *et al*, 2000). We found that high expression of MMP-1 by fibroblast cells correlated with the occurrence of metastasis, which is in accordance with previous studies showing that this MMP is associated with elevated metastasis capacity (Kang *et al*, 2003; Prybylowska *et al*, 2006). Matrix metalloproteinase -7 (matrilysin 1) is a stromelysin, that degrades type IV collagen, fibronectin and laminin. It was shown that MMP-7 is aberrantly expressed in human breast tumours and that elimination of MMP-7 is associated with low invasiveness and slow tumour growth (Jian *et al*, 2005). Likewise, it has been recently reported that MMP-7 overexpression in breast cancer (MCF-7) cells enhances cellular invasiveness and activation of proMMP-2 and MMP-9 (Wang *et al*, 2006). However, the potential role of MMP-7 in human breast cancer, and particularly in clinical breast cancer, has not been thoroughly investigated. Our results are in accordance with these experimental studies showing that high intratumoral levels of MMP-7 were significantly associated with several parameters indicatives de tumoral aggressiveness and linked with a high occurrence of distant metastasis.

Similarly to other studies, our data show that MMP-11 (Stromalysin-3) was preferentially expressed by peritumoral stromal cells (Basset *et al*, 1990; Basset *et al*, 1997) and that high levels of MMP-11 were associated with tumour progression and poor prognosis (Chenard *et al*, 1996; Ahmad *et al*, 1998). Matrix metalloproteinase -13 (collagenase-3) has been found to have an exceptionally wide substrate specificity when compared with other MMPs (Freije *et al*, 1994; Knauper *et al*, 1997). Moreover, it is thought to play a central role in the MMP activation cascade, both activating and being activated by several other MMPs (MMP-14, 2, or 3). Nielsen *et al* (2001) have reported that MMP-13 expression by myofibroblasts was often associated with microinvasive events, and they have proposed that this MMP may play an essential role during the transition of ductal carcinoma *in situ* lesions to invasive ductal carcinoma of the breast. In the present study, we found high MMP-13 expression in early-stage tumours, but also associated with tumours showing an infiltrating edge and with a higher rate of distant metastases when the MMP was expressed by fibroblastic cells or by inflammatory mononuclear cells. Matrix metalloproteinase -14 (membrane type 1 MMP, or MT1-MMP) is a key metalloprotease involved in the degradation of extracellular matrix, activates pro-MMP-13 (Knauper *et al*, 1996) and pro-MMP-2 (Strongin *et al*, 1995) on the cell surface, and plays crucial roles in molecular carcinogenesis, tumour cell growth, invasion, and angiogenesis. In the present study, we found significant associations between the expression of MMP-14 and clinicopathological parameters indicative of tumour aggressiveness. The strong association between MMP-14 expression by stromal cells and poor prognosis described in the present study is also remarkable .

Our results showing a significant association between TIMP-1, TIMP-2 and several parameters indicative of tumoral aggressiveness as well as with a high occurrence of distant metastases are in accordance with similar findings reported by other authors (Ree *et al*, 1997; Remacle *et al*, 2000; Schrohl *et al*, 2004). If TIMPs inhibit MMPs *in vivo*, it should be expected that high levels of these inhibitors would prevent tumour progression and thus be related with good outcome in patients with cancer. However, TIMPs are multifunctional proteins that in addition to their MMP-inhibitory effect also demonstrate distinct tumour-stimulatory functions (Jiang *et al*, 2002).

Experimental studies have shown that TIMP-3 may show activity to inhibit angiogenesis and induce apoptosis (Ahonen *et al*, 1998; Spurbeck *et al*, 2002). It has also been published that high TIMP-3 mRNA levels are associated with a good prognosis in breast cancer (Kotzsch *et al*, 2005). Likewise, Span *et al* (2004) have reported that high levels of TIMP-3 predicted a longer relapse-free survival in patients treated with tamoxifen. All these findings suggest that TIMP-3 is involved in specific pathways of tamoxifen-induced apoptosis. Our results show a significantly higher TIMP-3 expression in ER-positive tumors, in accordance with a prior study (Span *et al*, 2004). However, we also found that TIMP-3 expression by fibroblastic cells, but not by tumoural cells, correlates positively with the occurrence of distant metastases, reflecting the existence of other mechanisms in the molecular biology of the breast tumours in which this TIMP might be implicated.

In the present study, relevant our finding that the expression of MMP-1, 7, 11, 14, TIMP-1, 2 or 3 by fibroblasts and/or by inflammatory mononuclear cells was significantly associated with a higher incidence of distant metastases was especially , suggesting that the tumoral stroma does not play a merely passive role in cancer progression. In fact, over the past few years evidence has accumulated that both changes in stromal behaviour and the interaction between tumour cells and stromal cells are intimately linked to the processes of tumorgenesis, tumour invasion, and metastasis (Liotta and Kohn, 2001; Klausner, 2002; Wiseman and Werb, 2002). It has been demonstrated that several types of malignant cells (eg, breast and colon) actively recruit fibroblasts into tumours, leading to an increase in the extent of extracellular matrix degradation (Sloane *et al*, 2005). Likewise, it has been shown that incubation of breast cancer cells with monocytes or macrophages induces a crosstalk that results in an increased expression of factors involved in cancer cell invasiveness and in a modification of the monocytes function against cancer cells (Blot *et al*, 2003; Pukrop *et al*, 2006). We could hypothesise that tumours secrete factors able to elicit a wound-repair response from tumour-associated and tumour-infiltrating inflammatory cells, this response inadvertently stimulating tumour progression (Queen *et al*, 2005); or that it is the host tissue, with a redundant response of biochemical factors to cancerous cells, that induces the tumour growth. Even so, our data indicate a biological variability in the behaviour of these stromal cells with regard to the expression of MMPs and TIMPs, which is of clinical importance.

Our data also show that the expression of some MMPs and TIMPs has a potential value as predictor of distant metastases except for MMP-7 and MMP-14, without lymph node involvement. By contrast, we have surprisingly found that the global expression of TIMP-2 and MMP-13 correlated negatively with lymph node involvement. Altogether, our data support the concept that the biological mechanisms involved in blood vessel and lymphatic dissemination are dependent on different processes within tumoral pathophysiology. Nevertheless, we also have to consider that in the present study, we investigated the intratumoral stroma, and the putative absence of intratumoral lymphatics in invasive breast carcinomas is well known (Vleugel *et al*, 2004). On the other hand, it is also of note that we did not find well-defined cluster groups with regard to scores of immunostaining values of MMPs and TIMPs, which iis probably due to the biological heterogeneity of breast cancer.

In summary, our results demonstrate the importance of MMPs and TIMPs in the progression of breast cancer, and suggest their value in order to reach a more precise prognostic estimation in invasive ductal carcinoma of the breast.

## Figures and Tables

**Table 1:** Basal characteristics of 131 patients with invasive ductal carcinoma of the breast

	**Without recurrence**	**With recurrence**
**Characteristics**	**No. (%)**	**No. (%)**
Total cases	66 (100)	65 (100)
		
*Age (years)*		
⩽58	32 (48.5)	38 (58.5)
>58	34 (51.5)	27 (41.5)
		
*Menopausal status*		
Premenopausal	21 (31.8)	18 (27.7)
Postmenopausal	45 (68.2)	47 (72.3)
		
*Tumoural size*		
T1	36 (54.5)	27 (41.5)
T2	30 (45.5)	38 (58.5)
		
*Nodal status*		
N (−)	34 (51.5)	28 (43.1)
N (+)	32 (48.5)	37 (56.9)
		
*Histological grade* a		
Well Dif.	23 (34.8)	13 (20)
Mod. Dif.	29 (43.9)	35 (53.8)
Poorly Dif.	14 (21.2)	17 (26.1)
		
*Nottingham pronostic index*		
<3.4	29 (63)	17 (37)
3.4–5.4	28 (46.7)	32 (53.3)
>5.4	9 (36)	16 (64)
		
*Estrogen receptors* a		
Negative	26 (39.4)	36 (55.4)
Positive	40 (60.6)	29 (44.6)
		
*Progesterone receptors* a		
Negative	27 (37.9)	42 (64.6)
Positive	39 (59.1)	23 (35.4)
		
*Adjuvant radiotherapy*		
No	48 (72.7)	33 (50.8)
Yes	18 (27.3)	32 (49.2)
		
*Adjuvant systemic therapy*		
Chemotherapy	20 (30.3)	30 (46.2)
Adjuvant tamoxifen	26 (39.4)	15 (23.1)
Chemotherapy *plus* sequential tamoxifen	11 (16.7)	7 (10.8)
No treatment	9 (13.6)	14 (21.5)

a Criteria reported by Bloom and Richardson.

ER and PgR receptor measurements were performed on cytosol extracts by using a enzyme immunoassay (Monoclonal from Abbot Laboratories, Diagnostics Division, Wiesbaden, Germany). A value higher than 10 fmol mg^−1^ total protein was considered as positive.

**Table 2:** Relationship between MMPs and TIMPs immunostaining score values and clinico-pathological characteristics in 131 patients with invasive ductal carcinoma of the breast

**Characteristics**	**No.**	**MMP-1**	**MMP-2**	**MMP-7**	**MMP-9**	**MMP-11**	**MMP-13**	**MMP-14**	**TIMP-1**	**TIMP-2**	**TIMP-3**
Total cases	131	134 (0–285)	0 (246)	124 (0–270)	72 (0–273)	148 (0–279)	61 (0–234)	107 (0–261)	144 (0–285)	115 (0–243)	110 (0–272)
											
*Age (years)*											
⩽58	70	140 (20–285)	0 (0–207)	119 (0–270)	0 (0–264)	165 (0–279)	63 (0–180)	84 (0–261)	146 (0–285)	108 (0–243)	126 (0–272)
>58	61	140 (35–285)	0 (0–246)	145 (0–267)	72 (0–273)	147 (0–276.8)	59 (0–234)	81 (0–184.9)	136 (0–273)	118 (0–243)	75 (0–264)
											
*Menopausal status*											
Premenopausal	39	129 (20–285)	23 (0–207)	102 (0–270)	69 (0–176)	157 (0–277)	63 (0–180)	81 (0–261)	134 (0–285)	78 (0–231)	135 (0–261)
Postmenopausal	92	140 (27–285)	0 (0–246)	132 (0–267)	73 (0–273)	156 (0–279)	60 (0–234)	85 (0–258)	149 (0–282)	120 (0–243)	110 (0–272)
											
*Tumoral size*					*P*<0.05						
T1	63	131 (20–285)	0 (0–198)	119 (0–258)	65 (0–273)	144 (0–279)	66 (0–192)	81 (0–231)	134 (0–276)	78 (0–243)	116 (0–272)
T2	68	142 (27–285)	0 (0–246)	131 (0–270)	79 (0–264)	163 (0–277)	58 (0–234)	86 (0–261)	150 (0–285)	123 (0–243)	103 (0–271)
											
*Nodal status*				*P*<0.001			*P*<0.005				
N (−)	62	145 (20–285)	0 (0–207)	62 (0–243)	75 (0–273)	166 (0–279)	67 (0–234)	82 (0–261)	140 (0–276)	140 (0–243)	117 (0–272)
N (+)	69	140 (27–285)	0 (0–246)	151 (33–270)	70(0–237)	152 (0–276)	56 (0–192)	84 (0–258)	144 (0–285)	108 (0–243)	96 (0–265)
											
*Stage*				*P*<0.001			*P*<0.05				
I	39	139 (20–285)	0 (0–136)	62 (0–160)	69 (0–273)	152 (0–279)	67 (0–147)	81 (0–231)	134 (0–276)	77 (0–243)	118 (0–272)
II	61	140 (35–285)	0 (0–207)	132 (0–270)	76 (0–264)	161 (0–277)	62 (0–234)	85 (0–261)	148 (0–285)	118 (0–243)	117 (0–271)
III	31	132 (27–285)	0 (0–246)	157 (33–262)	68 (0–237)	147 (0–273)	54 (0–192)	81 (0–258)	146 (0–282)	120 (0–243)	72 (0–260.4)
											
*Histological grade*								*P*<0.05			
Well Dif.	36	140 (35–277)	0 (0–246)	70 (0–247)	67 (0–264)	103 (0–263)	66 (0–234)	78 (0–184)	144 (0–270)	79 (0–243)	117 (0–261)
Mod. Dif.	64	138 (20–285)	0 (0–207)	145 (0–258)	74 (0–273)	161 (0–279)	61 (0–192)	82 (0–261)	134 (0–276)	124 (0–243)	94 (0–271)
Poorly Dif.	31	145 (27–285)	0 (0–134)	120 (0–270)	96 (0–180)	172 (0–273)	57 (0–136)	121 (0–258)	151 (0–285)	74 (0–243)	94 (0–272)
											
*Nottingham pronostic index*				*P*<0.005							
<3.4	45	142 (20–285)	0 (0–136)	65 (0–239)	69 (0–273)	151 (0–279)	67 (0–234)	83 (0–261)	138 (0–276)	79 (0–243)	117 (0–261)
3.4–5.4	60	138 (33–285)	0 (0–246)	135 (0–267)	74 (0–237)	158 (0–277)	61 (0–192)	81 (0–255)	138 (0–276)	128 (0–243)	122 (0–272)
>5.4	25	140 (27–285)	47 (0–136)	139 (33–270)	82 (0–154)	159 (0–273)	56 (0–133)	147 (0–258)	146 (0–285)	85 (0–180)	71 (0–264)
											
*Oestrogen receptors*											*P*<0.01
Negative	62	136 (27–285)	19 (0–246)	130 (0–267)	73 (0–273)	152 (0–279)	58 (0–147)	85 (0–258)	134 (0–282)	120 (0–243)	72 (0–263)
Positive	69	140 (20–285)	0 (0–207)	120 (0–270)	72 (0–237)	160 (0–277)	63 (0–234)	81 (0–261)	146 (0–285)	90 (0–243)	137 (0–272)
											
*Progesterone receptors*											
Negative	69	135 (27–285)	0 (0–246)	129 (0–267)	75 (0–273)	155.4 (0–277)	56 (0–147)	85 (0–258)	146 (0–282)	122 (0–243)	91 (0–271)
Positive	62	140 (20–285)	0 (0–207)	126 (0–270)	70 (0–237)	160 (0–279)	63 (0–234)	81 (0–261)	136 (0–285)	72 (0–243)	118 (0–272)
											
*Desmoplastic reaction*						*P*<0.05			*P*<0.01		
No	45	130 (20–285)	0 (0–207)	129 (0–267)	66 (0–273)	112 (0–277)	52 (0–180)	81 (0–261)	119 (0–273)	78 (0–243)	95 (0–272)
Yes	86	140 (27–285)	0 (0–246)	127 (0–270)	75 (0–264)	164 (0–279)	62 (0–234)	84 (0–258)	151 (0–285)	125 (0–243)	116 (0–262)
											
*Peritumoral inflammation*			*P*<0.01					*P*<0.001			
No	82	140 (20–285)	0 (0–136)	120 (0–267)	69 (0–180)	152 (0–279)	61 (0–234)	79 (0–231)	137 (0–282)	112 (0–243)	121 (0–272)
Yes	49	134 (35–285)	47 (0–246)	130 (0–270)	75 (0–273)	161 (0–276.8)	60 (0–192)	154 (0–261)	150 (0–285)	118 (0–243)	74 (0–261)
											
*Tumoral advancing edge*							*P*<0.05				
Expansive	56	143 (20–285)	0 (0–136)	129 (0–262)	71 (0–264)	144 (0–279)	57 (0–180)	84 (0–261)	135 (0–276)	80 (0–243)	138 (0–272)
Infiltrating	75	140 (27–285)	0 (0–246)	125 (0–270)	75 (0–273)	164 (0–277)	63 (0–234)	82 (0–184)	150 (0–285)	124 (0–243)	81 (0–257)
											
*Vascular invasion*											
No	85	140 (20–285)	0 (0–207)	119 (0–267)	71 (0–273)	148 (0–279)	60 (0–192)	82 (0–255)	136 (0–282)	118 (0–243)	116 (0–272)
Yes	46	136 (40–285)	0 (0–246)	142 (0–270)	74 (0–174)	164 (0–265)	63 (0–234)	84 (0–261)	149 (0–285)	111 (0–243)	100 (0–264)

Data are expressed as median (range).

Statistical results were corrected applying Bonferroni's correction.

Samples on tissue sections were either insufficient or lost for analysis in three cases for MMP1, two cases for MMP-2, four cases for MMP-7, one for MMP-9, two for MMP-11, one for TIMP-1 and one for TIMP-2. The values shown correspond to the total of cases analyzed for each protein.

**Table 3:** Cox's univariate (HR) and multivariate (RR) analysis of the relationship between MMPs and TIMPs expression and relapse-free survival

**Factor**	**No. of patients**	**Event frequency**	**HR (95% CI)**	**RR (95% CI)**
*MMP-1*				
Score <median *vs* >median	66/62	29/36	1.6 (0.9–2.6)	—
Tumoural cells (−) *vs* (+)	15/113	4/61	2.5 (0.9–6.9)	—
Fibroblasts (−) *vs* (+)	27/101	9/56	2.1 (1–4.3)^****^	—
MIC (−) *vs* (+)	43/85	18/47	1.6 (0.9–2.9)	—
				
*MMP-2*				
Score <median *vs* >median	76/53	38/27	1 (0.6–1.7)	—
Tumoral cells (−) *vs* (+)	86/43	41/24	1.2 (0.7–2.1)	—
Fibroblasts (−) *vs* (+)	98/31	47/18	1.3 (0.7–2.3)	—
MIC (−) *vs* (+)	127/2	69/1	0.8 (0.1–6.1)	—
				
*MMP-7*				
Score <median *vs* >median	66/61	37/28	0.8 (0.5–1.4)	—
Tumoural cells (−) *vs* (+)	16/111	Jun-59	1.6 (0.7–3.7)	—
Fibroblasts (−) *vs* (+)	37/90	15/50	1.8 (1–3.2)^***^	1.8 (1–3.2)^****^
MIC (−) *vs* (+)	64/63	27/38	1.6 (1–2.7)^***^	—
				
*MMP-9*				
Score <median *vs* >median	65/65	20/44	2.7 (1.6–4.7)^*^	2.6 (1.5–4.5)^*^
Tumoural cells (−) *vs* (+)	35/95	7/57	3.7 (1.7–8.2)^*^	3.3 (1.5–7.4)^**^
Fibroblasts (−) *vs* (+)	110/20	44/20	3.7 (2.2–6.4)^*^	3.1 (1.8–5.3)^*^
MIC (−) *vs* (+)	116/19	50/14	3.6 (2–6.8)^*^	3 (1.6–5.5)^*^
				
*MMP-11*				
Score <median *vs* >median	66/63	24/40	2.1 (1.3–3.6)^**^	2.5 (1.5–4.3)^*^
Tumoural cells (−) *vs* (+)	15/114	4/60	2.1 (0.7–6.0)	—
Fibroblasts (−) *vs* (+)	41/88	7/57	5.7 (2.6–12.7)^*^	4.5 (2–10.1)^*^
MIC (−) *vs* (+)	89/40	25/39	6.0 (3.5–10.1)^*^	4.5 (2.6–7.7)^*^
				
*MMP-13*				
Score <median *vs* >median	66/65	35/30	0.7 (0.4–1.1)	—
Tumoural cells (−) *vs* (+)	34/97	17/48	0.8 (0.5–1.5)	—
Fibroblasts (−) *vs* (+)	67/64	24/41	2.0 (1.2–3.4)^***^	1.9 (1.1–3.2)^***^
MIC (−) *vs* (+)	87/44	35/30	2.1 (1.3–3.5)^**^	2.2 (1.3–3.7)^**^
				
*MMP-14*				
Score <median *vs* >median	66/65	28/37	1.4 (0.9–2.4)	—
Tumoural cells (−) *vs* (+)	13/118	5/60	1.4 (0.5–3.3)	—
Fibroblasts (−) *vs* (+)	25/106	11/54	1.1 (0.6–2.2)	—
MIC (−) *vs* (+)	64/67	16/49	4.8 (2.7–8.8)^*^	4.4 (2.4–8.1)^*^
				
*TIMP-1*				
Score <median *vs* >median	65/65	26/38	2.0 (1.2–3.3)^**^	1.7 (1.02–2.8)^****^
Tumoural cells (−) *vs* (+)	8/123	3/62	1.8 (0.5–5.8)	—
Fibroblasts (−) *vs* (+)	67/64	36/29	0.8 (0.4–5.8)	—
MIC (−) *vs* (+)	98/33	37/28	2.8 (1.7–4.6)^*^	2.2 (1.3–3.7)^*^
				
*TIMP-2*				
Score <median *vs* >median	65/65	17/47	3.7 (2.1–6.5)^*^	3.1 (1.7–5.5)^*^
Tumoural cells (−) *vs* (+)	21/110	3/62	4.8 (1.5–15.4)^***^	3.6 (1.1–11)^****^
Fibroblasts (−) *vs* (+)	76/55	18/47	7.1 (4.0–12.5)^*^	5.7 (3.2–10.1)^*^
MIC (−) *vs* (+)	81/50	25/40	3.4 (2.1–5.7)^*^	3.4 (2–5.7)^*^
				
*TIMP-3*				
Score <median *vs* >median	66/65	31/39	0.9 (0.6–6.5)	—
Tumoural cells (−) *vs* (+)	18/113	9/56	1 (0.5–2)	—
Fibroblasts (−) *vs* (+)	51/80	16/49	2.4 (1.4–4.3)^**^	2 (1.1–3.7)^***^
MIC (−) *vs* (+)	62/69	28/37	1.3 (0.7–2)	—

Abbreviations: CI=confidence interval; HR= hazard ratio; MIC=mononuclear inflammatory cells; RR= relative risk.

^*^P<0.001; ^**^P<0.005; ^***^P<0.01; ^****^P<0.05.
